# Vector Auto-Regression-Based False Data Injection Attack Detection Method in Edge Computing Environment

**DOI:** 10.3390/s22186789

**Published:** 2022-09-08

**Authors:** Yi Chen, Kadhim Hayawi, Qian Zhao, Junjie Mou, Ling Yang, Jie Tang, Qing Li, Hong Wen

**Affiliations:** 1 College of Electronic Engineering, Chengdu University of Information Technology, Chengdu 610225, China; 2 School of Aeronautics and Astronautics, University of Electronic Science and Technology of China, Chengdu 611731, China; 3 CMA Key Laboratory of Atmospheric Sounding, Chengdu 610225, China; 4 College of Technological Innovation, Zayed University, Abu Dhabi 144534, United Arab Emirates

**Keywords:** false data injection attack (FDIA), vector auto-regression (VAR), attack detection, smart grid

## Abstract

With the wide application of advanced communication and information technology, false data injection attack (FDIA) has become one of the significant potential threats to the security of smart grid. Malicious attack detection is the primary task of defense. Therefore, this paper proposes a method of FDIA detection based on vector auto-regression (VAR), aiming to improve safe operation and reliable power supply in smart grid applications. The proposed method is characterized by incorporating with VAR model and measurement residual analysis based on infinite norm and 2-norm to achieve the FDIA detection under the edge computing architecture, where the VAR model is used to make a short-term prediction of FDIA, and the infinite norm and 2-norm are utilized to generate the classification detector. To assess the performance of the proposed method, we conducted experiments by the IEEE 14-bus system power grid model. The experimental results demonstrate that the method based on VAR model has a better detection of FDIA compared to the method based on auto-regressive (AR) model.

## 1. Introduction

The stable and reliable operation of power system is very important for all walks of life [1,2,3,4]. The function of power system state estimation is to estimate the current operation state of power system according to various measurement information of power system. Accurate power system state estimation is conducive to the reliable operation and real-time control of power system [5,6,7]. It enables the management system to perform various important control and planning tasks, such as emergency analysis, voltage stability studies and optimized power flow analysis [8]. Therefore, power system security is extremely important.

With the development of smart grid, the efficiency and reliability of power system are gradually improving, at the same time, the power system is also facing the potential risk of network attack. In the past twenty years, the power grid has been subjected to major security threats several times [9,10,11,12]. On 25 January 2016, the Israeli power system was subjected to a massive cyber attack intrusion due to the unintentional execution of malicious code by staff members, triggering the urgent removal of a large number of power industrial control computers from operational status [13]. In June 2018, hackers successfully attacked a French company, named as Ingerop, and successfully stole confidential documents related to the nuclear power plant, which made the nuclear power plant and its staff expose to the threat of terrorist plots [11]. In September 2020, Pakistan’s largest power supplier, K-Electric, was attacked by blackmail software and stolen unencrypted files. This attack directly led to the interruption of billing and online services, resulting in the supplier’s customers being unable to access the online resources of their accounts [12].

For resisting network attacks, researchers have proposed a lot of security schemes, such as firewalls and conventional intrusion detection systems [14,15,16,17], false data detection system [18]. In the false data attacks, there are mainly two categories of research on constructing false data for injection attack [19]: one is that the attacker previously obtains the network topology information of the target for attack; the other is that the attacker has no the topology information of the target for attack in advance, but constructs the attack vector with the only intercepted measurements information.

For the first category of attack methods, one of the representative methods is the false data injection attack (FDIA) method proposed by Liu against power grid state estimation [20]. The attacker distorts the measurement data collected by the supervisory control and data acquisition (SCADA) system. That is, the attacker tampers with the measurement data collected by the intelligent terminal, which would destroy the data availability and integrity in power networks [21,22]. In addition, the literature [23,24] introduce an in-depth study on how to attack while the attacker has information about the target of the attack. However, the information of the target to be attacked is usually in a confidential state, so the possibility of obtaining this information is small.

As for the second type of attack methods, the representative literatures are [25,26], in which they present the singular value decomposition (SVD) and the principal component analysis (PCA) based attack vector construction methods, respectively. Compared with the first category of attacks, the second category of attacks is more feasible and poses a greater threat to the smart grid system. Nevertheless, the efforts made specifically for the smart grid are very limited against the network security.

In addition, due to the proximity of the attacker to the endpoints, malicious attacks on the terminals will be easier to launch. If the attackers know the topology of the smart grid, they are easy to construct the FDIA vector without changing the measurement residuals and thus affect the overall system state estimation. So, detection and identification of bad data is very important for the computing center [27,28,29]. In the actual system, the measurement data of the terminal is collected from the field and then transmitted to the control center. Unfortunately, the attacker’s FDIA behavior may be ignored by the bad data detection (BDD) system. This will pose a threat to the state estimation of the smart grid and affect the smart grid decisions [30].

Previous works have introduced several methods to detect false data injection attacks. For instance, Liu et al. proposed a false data detection mechanism based on the separation of nominal power grid states and anomalies [31]. Li et al. presented a method of detecting FDIAs against power system state estimation with fast Go-decomposition approach [32]. Zhao et al. introduced an FDIA dectection method based on short-term state forecasting by checking the statistical consistency between forecasted and gathered measurements [33]. Ashok et al. put forward an online FDIA detection method that availed of load forecasts, generation schedules, and synchrophasor data to detect measurement anomalies [34]. In addition, there are also several FDIA detection methods based on AC state estimation. For example, Du et al. [35] proposed a FDIA model against nonlinear state estimation by leveraging the intrinsic load dynamics inside the attacking region and drawing upon the regression theorem of the Ornstein–Uhlenbeck process and weighted least square estimations. Boyaci et al. [36] presented a FDIA detector based on graph neural network (GNN) by incorporating the inherent physical connections of modern AC power grids and exploiting the spatial correlations of the measurement. Cheng et al. [37] put forward a false data injection attack detector named the *k*-smallest residual similarity test based on the rationale that perfect false data injection attacks can hardly be achieved in AC state estimation. Although the nonlinear AC model based FDIA detection methods have been proved to be safer than the DC model based methods in most occasions of power system, the linear DC state estimation model based methods are still widely applied to the power system because of their linear expression and rapidity.

Besides, smart grid under edge computing architecture has many advantages, such as low latency, high speed, high reliability, and high security [38,39,40,41]. The edge devices usually have higher capacity than the intelligent terminals. Making full use of the computation power and memory of edge computing to implement FDIA detection is very important and practical for the development and safe operation of smart grid. For the FDIA detection, there are three main directions, which are before, during, and after the completion of state estimation based on the detection time. Most of the current detection methods are the “after state estimation”. However, the detection time of the detection scheme that works best, in theory, should be before the state estimation. This will minimize the effect of spurious data on the state estimation results. Therefore, this paper proposes a method of FDIA detection based on VAR to detect spurious data before state estimation in edge computing environment. Thus, it is conducive to the safe and stable operation of smart grid.

The contributions of this paper are summarized as follows:

(1) Develop a VAR-based FDIA detection method, aiming to improve safe operation and reliable power supply for smart grid applications in terms of accuracy of voltage phase prediction state and FDIA detection rate.
(2) Introduce the FDIA detection procedure of the proposed method in detail. The proposed method is characterized by incorporating with VAR model and measurement residual analysis based on infinite norm and 2-norm to achieve the FDIA detection under the edge computing architecture.
(3) Launch different simulation via IEEE 14-bus system to verify the proposed VAR-based FDIA detection method. The results indicate that the proposed method is more efficient than the comparison.

The remainder of the paper is organized as follows. Section 2 describes the background knowledge of vector auto-regression. Section 3 illustrates the FDIA model of the study. Section 4 introduces the proposed VAR-based FDIA detection method. The performance evaluation and analysis of the proposed method is given by Section 5. Finally, the paper is concluded in Section 6.

## 2. Background

### Vector Auto-Regression

Vector auto-regression (VAR) is a statistical model used to capture the relationship between multiple variables as they change over time. It is an extension of auto-regression (AR) model. Like the auto-regressive model, each variable in the VAR model has an equation modelling its evolution over time. The specific form of VAR is expressed as Equation (1):

(1) $$x_{k}=T_{k-1}\times x_{k-1}+\cdots +T_{k-p}\times x_{k-p}+\varepsilon_{k}$$

where *x* represents variable vector, *k* denotes time, and *p* is a constant. Compare to $x_{k}$, the $x_{k-1}$,⋯, $x_{k-p}$ are the variable vector from lag phase 1 to lag phase *p*, respectively. *T* is a time-invariant parameter matrix. $\varepsilon_{k}$ is an interference error term at *k* time.

Vector auto-regression model is widely used in economics and the natural sciences [42]. Literature [43] uses VAR model with graph regularization to predict microbial interactions. Literature [44] studies the causal relationship between rainfall and temperature by simultaneously constructing and predicting bivariate VAR model. H. Wang et al. utilize time-varying vector auto-regressive model to recognize the multi-task motor imagery EEG (electroencephalogram) signals in literature [45]. It verifies that the time-varying vector auto-regressive model is useful to analyze autocovariance nonstationary vector process. Inspired by the previous application of VAR, we propose a VAR-based FDIA detection method for the smart grid application under edge computing structure.

## 3. FDIA Model

**Figure 1:**
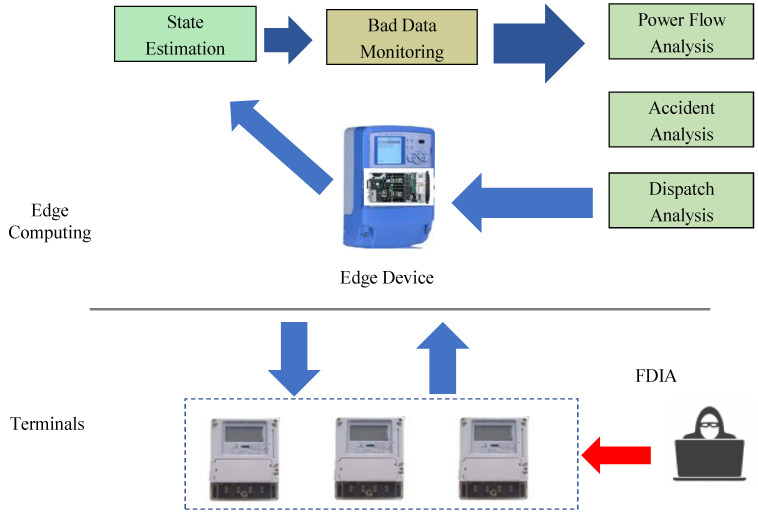
FDIA model under edge computing.

This section introduces the FDIA model in edge computing environment, which is shown in Figure 1. The attacker launches FDIA on the data collected from the intelligent power terminal lines, thereby affecting the state estimation of edge devices. However, the bad data detection (BDD) system could not detect the FDIA, thus the decision-making system will make wrong decisions on such as the power flow analysis, accident analysis, economic dispatch analysis and so on. The FDIA is modeled as Equation (2):

(2) $$\tilde{z}=H\times \hat{x}+v+a$$

where *H* denotes the measurement Jacobian matrix, which is determined by the structure of the system; $\hat{x}$ presents the state estimation of power system; *v* is the measurement error; and $a\in R^{N}$ is an attack vector.

Generally, the attacker randomly selects a non-zero vector and calculates the attack vector *a* shown as Equation (3):

(3) a=H×c

where $c={\left(c_{1},c_{2},\cdots ,c_{n}\right)}^{T}$ is an any non-zero *n*-dimensional vector.

After adding the attack vector *a*, the new state estimation vector is presented as Equation (4):

(4) $$\begin{array}{cc} {\hat{x}}_{a} & ={\left(H^{T}\times R^{-1}\times H\right)}^{-1}\times H^{T}\times R^{-1}\times z_{a}\\ \; & =\hat{x}+c \end{array}$$

where *R* denotes attack matrix, $R^{-1}$ is the inverse of *R*, and $z_{a}$ is the measured value after being attacked.

Then, the residual error $r_{a}$ is shown as Equation (5):

(5) $$\begin{array}{cc} r_{a} & =z_{a}-H\times {\hat{x}}_{a}\\ \; & =z+a-H\times (\hat{x}+c)\\ \; & =z-H\times \hat{x}+\left(a-H\times c\right)\\ \; & =z-H\times \hat{x}\\ \; & =r \end{array}$$

## 4. FDIA Detection Method Based on Vector Auto-Regression

This section mainly introduce the FDIA detection method based on vector auto-regression model in edge computing environment.

If the Vector auto-regression model is set as p=1 [46], it means that the samples at the first *k* time are used to predict the state at k+1 time. When the linear state estimation is employed, the voltage phase angle of the state estimation vector *x* is θ, and the voltage amplitude *V* is always 1, the prediction model of system state is modeled as Equation (6):

(6) $${\tilde{\theta }}_{k+1}=T_{k}\times {\hat{\theta }}_{k}+\varepsilon_{k+1}$$

where ${\tilde{\theta }}_{k+1}$ and $T_{k}$ are predicted state voltage phase angle and parameter matrix, respectively. *k* and k+1 are sampling time. ${\hat{\theta }}_{k}$ represents the voltage phase angle obtained from the state estimation at *k* time, whose dimension is n×1. $\varepsilon_{k+1}$ is the error of system model and the Gaussian white noise with zero mean.

Then, we obtain the covariance matrix $R_{{\tilde{x}}_{k+1}}$ of state prediction voltage phase angle vector ${\tilde{\theta }}_{k+1}$ by calculating the mathematic expectation on both sides of Equation (6) at the same time. The covariance matrix is shown as follow:

(7) $$R_{{\tilde{\theta }}_{k+1}}=T_{k}\times R_{{\hat{\theta }}_{k}}\times T_{k}^{T}+R_{\varepsilon_{k+1}}$$

(8) $$R_{{\hat{\theta }}_{k}}=E\left[\left(\theta_{k}-{\hat{\theta }}_{k}\right)\cdot {\left(\theta_{k}-{\hat{\theta }}_{k}\right)}^{T}\right]$$

(9) $$R_{\varepsilon_{k+1}}=E\left(\varepsilon_{k}\times \varepsilon_{k+1}\right)$$

where $R_{{\hat{\theta }}_{k}}$ is the state prediction error matrix at time *k*, which is usually assumed to be a normal distribution. ${\hat{\theta }}_{k}$ is the vector of state estimates at the time sample *k* before. E(·) is the expectation operator. Because $R_{{\hat{\theta }}_{k}}$ and $\varepsilon_{k}$ obey normal distribution, it is easy to prove that $R_{{\tilde{\theta }}_{k+1}}$ obeys normal distribution. Therefore, the predicted value of measured active power ${\tilde{P}}_{k+1}$ at time sample point k+1 can be calculated from the predicted value of state voltage phase angle ${\tilde{\theta }}_{k+1}$, ${\tilde{P}}_{k+1}$ is expressed as Equation (10):

(10) $${\tilde{P}}_{k+1}=H\times {\tilde{\theta }}_{k+1}$$

The prediction error covariance matrix is expressed as Equation (11):

(11) $$\begin{array}{cc} Cov(\tilde{P}) & =H\times Cov\left(\tilde{\theta }\right)H^{T}\\ \; & =H\times R_{\tilde{\theta }}\times H^{T} \end{array}$$

To simplify equations, Equation (11) and all the following equations omit the time index. Residual of the measured observed and predicted values are obtained in Equation (12):

(12) $$\tilde{r}=\tilde{P}-P$$

In theory, the residual $\tilde{r}$ follows the Gaussian distribution with a mean value of 0 and covariance matrix of *N*, where *N* is obtained by Equation (13).

(13) $$N=R+H\times R_{\tilde{\theta }}\times H^{T}$$

The measurement residual analysis method based on $L_{2}$ norm has been used in the control center for many years [47], and it has been proved to have good performance in dealing with bad data. Inspired by this, this paper innovatively proposes an enhanced and efficient FDI attack detection method, which is integrated the measurement residual analysis method based on *∞* norm and $L_{2}$ norm into the FDIA attack detector shown as Equation (14):

(14) $$D\left(z\right)=\left\{\begin{array}{cc} 1, & ∥P-H\hat{\theta }{∥}_{2}⩾\tau_{1}\;\mathrm{or}\;{∥\frac{\tilde{P}-P}{\sigma_{N}}∥}_{\infty }⩾\tau_{2}\\ 0, & other \end{array}\right.$$

where $\sigma_{N}=diag\left(N\right)$. If D(z)=1, it indicates that there is the FDIA; otherwise D(z)=0, it indicates that there is no FDIA. There are two thresholds $\tau_{1}$ and $\tau_{2}$ in the detector, which indicate the significance level of the hypothesis test. In the existing detector-based integration methods, the traditional residual-based bad data detection method, that is, the detection threshold $\tau_{1}$ remains the same, and $\tau_{1}$ is fixed to obtain the required error warning probability. The detection threshold $\tau_{2}$ as an alternative method is changed to test the performance of the detector. The time correlation under the normal operation state of the system shows that the measurement difference between the predicted measurement value and the observed measurement value should be consistent. Once the false injection data is applied to the measurement value, the consistency will be destroyed, so that the attack behavior can be detected.

If there is no FDIA, the state estimation result is reliable. Otherwise, detect the FDIA and process the measured value of the attacked, so as to re-estimate the state and obtain accurate estimation results. One way to deal with these attacked metrics is to delete them from the set of metrics so that they will not affect the final state estimation results. However, removing the attacked measurements may make the system unobservable. Here, the predicted measurements are used to replace these attacked measurements, and then the linear state estimation based on the mixing quantity is carried out to obtain a new and accurate system operation state. On the other hand, the predicted measurements can be further used as pseudo measurements to improve the observability of the system. In order to analyze and evaluate the prediction performance more intuitively, literature [33] uses the prediction method based on autoregressive model as a comparison. The attack vector is generated based on a random false data injection attack scheme. State vector ${\hat{\theta }}_{a}$ is updated from $\hat{\theta }$ to $\hat{\theta }+c$. Each row vector of *c* vector is randomly generated by Gaussian distribution, and the variance is $\sigma_{c}^{2}$. The value of $\sigma_{c}^{2}$ is determined by the signal-to-noise ratio. Here, the signal-to-noise ratio is specified as 10 dB, that is, SNR=10. The definition of the signal-to-noise ratio is expressed as Equation (15):

(15) $$SNR=10log\left(\frac{\sigma_{\theta_{a}}^{2}}{\sigma_{n}^{2}}\right)$$

(16) $$\sigma_{\theta_{a}}^{2}=\sigma_{\theta }^{2}+\sigma_{c}^{2}$$

where $\sigma_{\theta_{a}}^{2}$ and $\sigma_{\theta }^{2}$ denote the variance of each component of $\theta_{a}$ and θ, respectively.

## 5. Experimental Analysis

In this section, we firstly present our experimental overall setting in Section 5.1. Secondly, the evaluation index of the proposed method is introduced in Section 5.2. Finally, experimental results analysis are also demonstrated in Section 5.3.

### 5.1. Experimental Parameter Setting

**Figure 2:**
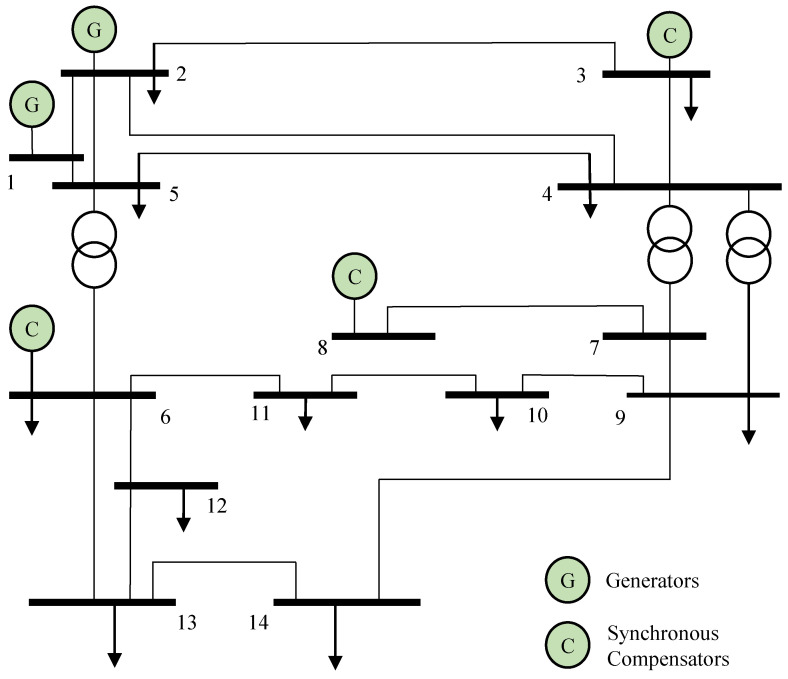
Power grid model of IEEE 14-bus system.

In the experiment, we simulate the structure of the power grid through the IEEE 14-bus system power grid model, as shown in Figure 2 [48,49]. First, we use the VAR model for short-term forecasting. Then, the difference between predicted data and observed values is detected by using a classification detector. Finally, the results of above state prediction and detection are evaluated in comparison with the AR model.

We use MATPOWER [50] to generate the data of IEEE 14-bus system, including a topology matrix with corresponding parameters, status and measured values of the system. The formats of MATPOWER bus data and branch data are shown in Table 1 and Table 2, respectively.

Here, the bus data is represented by a large matrix, and each line corresponds to a single bus. “bus_i” represents bus number, “type” represents bus type, “$P_{d}$” represents active power, “$Q_{d}$” represents reactive power of the load, “$G_{s}$” represents conductance in parallel with a single node, generally set to 0. “$B_{s}$” represents susceptance in parallel with a single node, generally set to 0. “area” represents bus section number, generally set to 1. “$V_{m}$” represents initial voltage amplitude, “$V_{a}$” represents initial voltage phase angle, “baseKV” represents reference voltage, “zone” represents bus loss saving area, and “$V_{max}$” represents maximum acceptable voltage, “$V_{min}$” represents minimum acceptable voltage. Field “branchdata” represents a matrix for setting parameters of each branch in power grid. Each line corresponds to a single branch. “fbus” represents starting node number of the branch, “tbus” represents ending node number of the branch. “*r*”, “*x*” and “*b*” are resistance, reactance and charging charge of the branch, respectively. “rateA”, “rateB” and “rateC” are long-term, short-term and emergency allowable power of the branch, respectively. “ratio” represents transformation ratio of the branch, and “angle” represents the phase angle of the branch. “status” represents working state of the branch, 1 represents input, and 0 represents exit. “$ang_{min}$” and “$ang_{max}$” represents the minimum and maximum difference of the phase angle of the branch, respectively.

In terms of the attacker’s access to information, it can be divided into access to global information [51] and local information [52]. In the experiment, we assume that the attacker has access to limited real-time data for online state estimation. Firstly, we make a linear approximation to an AC optimal power flow model. After that, it is transformed into a DC optimal power flow model for linear state estimation. Finally, a false data injection attack is simulated for the DC optimal power flow model. In this experiment, state quantity *x* and measured value *z* include the voltage phase angle θ and active power *P*, respectively. In addition, the voltage amplitude is set to V=1. It is assumed that the attacker has full knowledge of the target system’s topology and destroys the state estimation by injecting false data into the device. The measured value *z* in this paper is obtained by z=P+v. The error of the measured value collected by the data acquisition system usually follows the Gaussian distribution, which has a variance σ between 0.005 and 0.02 of the measured value without noise. We set the variance to 0.01 of the normal measured value. The attack vector used in this paper is generated by a random false data injection scheme. In the case of FDIA, attack nodes are randomly selected. In addition, the attack lasts for 20 min.

### 5.2. Evaluation Index

To evaluate the prediction performance of the detection scheme proposed in this paper, the mean square error (MSE) is used as the performance index for comparison. The MSE function is shown as follow:

(17) $$MSE=\frac{1}{M}\sum_{k=1}^{M}e_{k}^{2}$$

(18) $$e_{k}=\theta_{k}-{\tilde{\theta }}_{k}$$

where $\theta_{k}$, ${\tilde{\theta }}_{k}$ and *M* are observed state voltage phase angle values, predicted state voltage phase angle values of k-time sampling point and the number of time points, respectively.

To analyze the detection performance, the receiver operating characteristic (ROC) curve is used for evaluation. The ROC curve depicts the relative balance relationship between false-positive rate and true-positive rate.

The true positive rate is the proportion of false data detected out of the total false data, which is referred to as the probability of detection in this paper and expressed as Equation (19):

(19) $$P_{d}=\frac{N_{Hit}}{N_{Hit}+N_{Miss}}$$

The false positive rate is the proportion of all normal data that are falsely detected as being false, and is expressed as Equation (20):

(20) $$P_{f}=\frac{N_{Flase}}{N_{Flase}+N_{Correct}}$$

The false-alarm rate is the percentage of all false data that are falsely detected as normal data, and is expressed as Equation (21):

(21) $$P_{m}=\frac{N_{Miss}}{N_{Flase}+N_{Correct}}$$

Among them, $N_{Hit}$ is the number of true-positive successfully detected by false data. $N_{Miss}$ is the number of false-negative not detected by false data. $N_{False}$ is the number of false-positive wrongly detected by normal data, and $N_{Correct}$ is the number of true-negatives normally detected by normal data. The ideal situation is that the false positive rate is inversely proportional to the detection probability, that is, the lower the false positive rate, the higher the detection accuracy. This is because the false positive rate has a great impact on the power grid decision-making. The measured data increases continuously due to the growth of time, and the impact of the false positive rate increases in geometric multiples. Therefore, the final goal of the experiment is to achieve the $P_{c}$ highest value and the $P_{f}$ lowest value.

The attacker may have limited privileges, a limited budget, or the control center may have encrypted the historical data so that it is not available. In this paper, we assume that the attacker can only access a limited amount of real-time data for online state estimation, make a linear approximation of the AC tidal model, transform it into a DC tidal model for linear state estimation, and simulate a false data injection attack for the DC tidal model. The experiments in this paper use a state quantity *x* containing the voltage phase angle θ, a measurement z containing the active power *P*, and a constant voltage magnitude *V* of 1. It is assumed that the attacker knows the full structure of the Jacobi matrix *H*, i.e., has full knowledge of the topology of the target system, and corrupts the state estimate by feeding false data into the device. The measurement value *z* in this paper is obtained from z=P+v. The error of the measurement value collected by the supervisory control and data acquisition system usually obeys a Gaussian distribution, which has a variance σ of size 0.5% to 2% of the measurement value without noise. Thus, the variance σ is set to 0.01 of the normal measurement value in the experiment.

**Figure 3:**
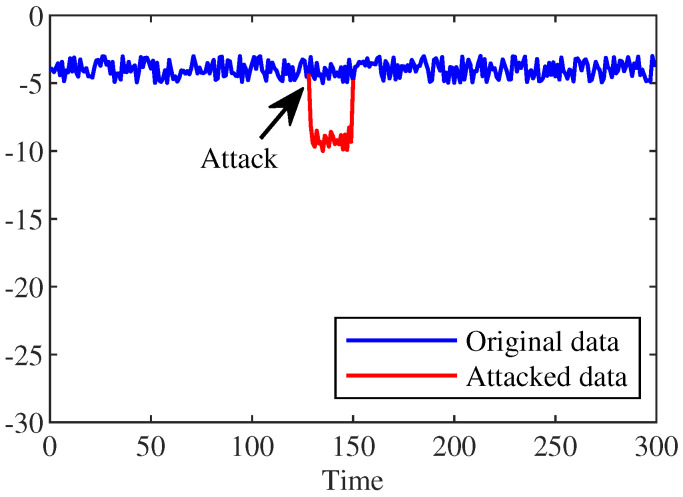
State change curve of sudden FDIA.

Figure 3 demonstrates the state change curve of one of the sudden false data injection attacks, where, in the case of sudden FDIA, the attack nodes are randomly selected and the attack lasts for 20 min. From Figure 3, we notice that the voltage phase angle values after the attack are very similar to the original voltage phase angle values. This makes the FDIA extremely stealthy, which makes it difficult to detect the attack by using common defense mechanisms.

### 5.3. Result Analysis

#### 5.3.1. Prediction Performance Analysis of VAR and AR Schemes

From Table 3, we find that the MSE of VAR-based method and AR-based method are 0.3613 and 1.5945, respectively. This indicates that the VAR-based scheme predicts better than the AR-based scheme. The results demonstrate that the data of each node in the grid is time-correlated, which is not only related to its own data on the current node but also related to the data on other nodes. Therefore, the prediction scheme should consider the correlation between nodes, which will improve the prediction performance.

#### 5.3.2. Analysis of Detection Performance of VAR and AR Schemes

Before analyzing the detection performance, we need to consider two things: one is whether the value of sliding window length *M* affects the detection effect, and the other is whether the number of non-zero values of non-zero vector *c* will affect the test results after participating in the construction of attack vector. In this regard, the following analysis is carried out.

(a) Influence of sliding window length *M*.

**Figure 4:**
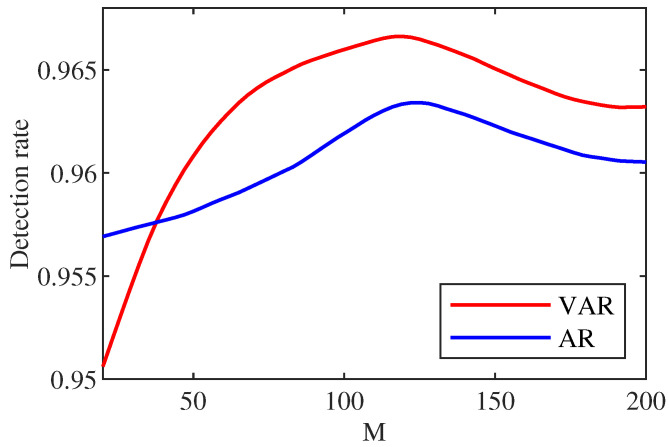
Comparison diagram of the relationship between *M* and detection rate under different methods.

Here, the influence of *M* on the detection rate is discussed. According to the control variable method, we set $P_{f}=0.05$ and the non-zero number in *c* is 2. Firstly, the state transfer matrix $T_{k}$ is calculated by updating *M*. Then, state prediction ${\tilde{\theta }}_{k+1}$ is calculated, followed by the calculation of measurement prediction ${\tilde{P}}_{k+1}$ to obtain the detection result. Finally, the detection rate is varied by changing the value of *M* to different detection rates. The experimental results are shown in Figure 4.

From Figure 4, we can notice that the detection performance of both schemes can be significantly improved at appropriate *M*, while inappropriate *M* value leads to deterioration of detection performance. The detection rate of the detection scheme predicted by the initial VAR scheme is not as high as that of the detection scheme predicted by the AR scheme. However, when specific limit values are exceeded, the detection rate of the prediction-based scheme using VAR is steadily higher than that of the AR scheme.

By analyzing the situation shown in Figure 4, we can draw the following conclusions.

In the case of linear prediction model, the accuracy of prediction is closely related to the state transition matrix of the model. The state transition matrix of the VAR model and AR model compared in this paper is related to redundancy *g*. Further, it is related to least square estimation. Because the least square estimation needs to calculate the coefficients of IEEE14 power system, and in the VAR model, we need to consider the influence of its own node and other nodes on this node, so the VAR model needs to calculate 13×13=169 coefficients, while the AR model only needs to calculate 13 coefficients. Even if the *M* value divided for power data is the same, the redundancy of the two models is different.

At the beginning, the initial value of sliding window length *M* is small, and the VAR model is not good enough, resulting in the imperfect prediction performance. The detection rate of the scheme predicted by VAR model is lower than that predicted by AR model. When the redundancy increases with the increase of *M*, the prediction accuracy of VAR model increases, and the detection rate of the corresponding detection scheme increases, which is higher than that of AR scheme. In the last stage, the redundancy is too high, which exceeds the computing power of the two regression models, so the value of *M* is 120 in the next simulation.

(b) Impact of the number of non-zero values in *c*.

**Figure 5:**
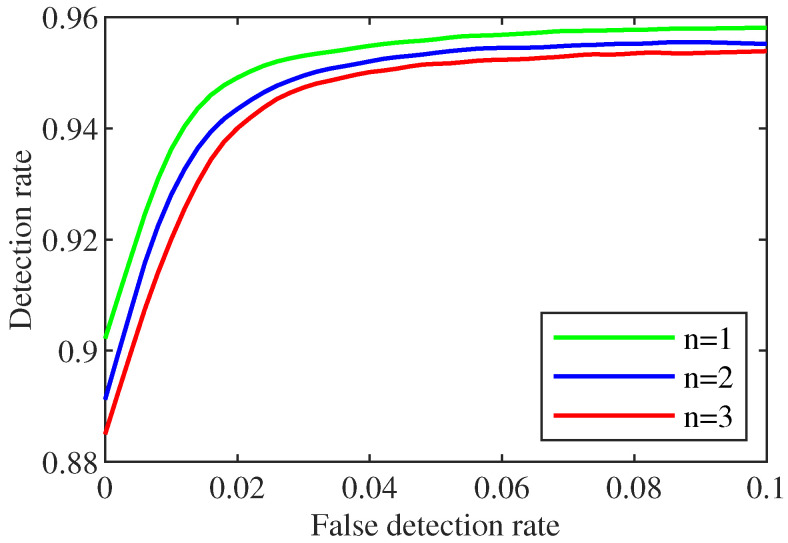
Comparison of the influence of non-zero quantity in *c* on detection performance.

From the above analysis (related Figure 4), we notice that a larger *M* value for the sliding window length is not better. Therefore, it is necessary to make a trade-off comparison between prediction accuracy and calculation speed. Here, the value of *M* is 120. This part discusses the influence of non-zero quantity in *c* on the detection rate. According to the control variable method, setting $P_{f}=0.05$. By changing the non-zero number in *c* to update the attack vector, a new state estimation value is obtained. The remaining steps are similar to the above (a), and different detection rate is shown in Figure 5.

Figure 5 shows the relationship between the different numbers of non-zero and the detection rate, where *n* is the number of non-zero. From Figure 5, we find that the early detection rate is proportional to the increase in *n*. The reason is that the increase in *n* leads to the increase of the number of non-zero in the attack vector, which increases the risk of measured value being attacked. This will greatly increase the difference between the observed and predicted value and facilitates control center to detect the attack.

After considering the impact of *M* and *c* on the detection rate, we set M=120 and the number non-zero of *c* to 2 in the experiment of attack detection.

**Figure 6:**
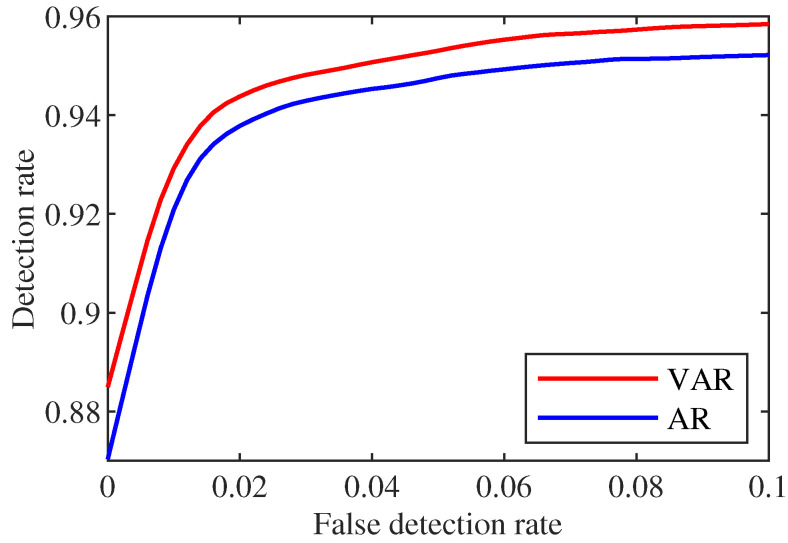
Comparison of detection rate and false detection rate under different method.

From Figure 6, we notice that VAR-based scheme prediction is very effective in detecting FDIA. Even if the false detection rate is very low, the detection rate can reach more than 0.87. For example, when the false detection rate is 0.05, the detection rate has reached 0.95. Moreover, the performance of the detection scheme using the VAR scheme prediction is better than the AR scheme in general. The reason is that with the current sliding window length *M* and a non-zero number of 2 for *c*, the redundancy is sufficient for the VAR model. Therefore, its prediction performance is better than the AR model and the classifier can detect better.

## 6. Conclusions

This paper focuses on the FDIA and innovatively proposes a VAR-based FDIA detection method for the smart grid application, which is integrated the measurement residual analysis method based on *∞* norm and $L_{2}$ norm into the FDIA attack detector. this paper proposes an enhanced and efficient FDI attack detection method. Then, We also introduce the proposed method in detail. The VAR model is used for prediction, and classifier is used for FDIA detection. Finally, we conduct the FDIA simulation experiments by computer. The results show that the proposed FDIA detection method based on VAR is better than the AR-based method in FDIA detection rate. Even when the false detection rate is low, the detection rate is higher than 0.87.

In the follow-up work, we will consider the effects of false data injection attack on different models and methods, and compare them with the proposed FDIA detection scheme based on VAR in this paper. In addition, we think that it is meaningful to analyze the impact of different noises in different scenarios on the performance of the FDIA detection scheme. Furthermore, it is also worthwhile to find new and effective measures to enhance the capability of defense system for smart grid.

## Figures and Tables

**Table 1:** Bus data format of IEEE 14-bus system.

bus_i	Type	$P_{d}$	$Q_{d}$	$G_{s}$	$B_{s}$	area	$V_{m}$	$V_{a}$	baseKV	zone	$V_{\max}$	$V_{\min}$
1	3	0	0	0	0	1	1.06	0	0	1	1.06	0.94
2	2	21.7	12.7	0	0	1	1.045	−4.98	0	1	1.06	0.94
3	2	94.2	19	0	0	1	1.01	−12.72	0	1	1.06	0.94
4	1	47.8	−3.9	0	0	1	1.019	−10.33	0	1	1.06	0.94
5	1	7.6	1.6	0	0	1	1.02	−8.78	0	1	1.06	0.94
6	2	11.2	7.5	0	0	1	1.07	−14.22	0	1	1.06	0.94
7	1	0	0	0	0	1	1.062	−13.37	0	1	1.06	0.94
8	2	0	0	0	0	1	1.09	−13.36	0	0	1.06	0.94
9	1	29.5	16.6	0	19	1	1.056	−14.94	0	1	1.06	0.94
10	1	9	5.8	0	0	1	1.051	−15.1	0	1	1.06	0.94
11	1	3.5	1.8	0	0	1	1.057	−14.79	0	1	1.06	0.94
12	1	6.1	1.6	0	0	1	1.055	−15.07	0	1	1.06	0.94
13	1	13.5	5.8	0	0	1	1.05	−15.16	0	1	1.06	0.94
14	1	14.9	5	0	0	1	1.036	−16.04	0	1	1.06	0.94

**Table 2:** Branch data format of IEEE 14-bus system.

fbus	tbus	*r*	*x*	*b*	rateA	rateB	rateC	ratio	angle	status	${\mathrm{ang}}_{\min}$	${\mathrm{ang}}_{\max}$
1	2	0.01938	0.05917	0.0528	9900	0	0	0	0	1	−360	360
1	5	0.05403	0.22304	0.0492	9900	0	0	0	0	1	−360	360
2	3	0.4699	0.19797	0.0438	9900	0	0	0	0	1	−360	360
2	4	0.05811	0.17632	0.034	9900	0	0	0	0	1	−360	360
2	5	0.05695	0.17388	0.0346	9900	0	0	0	0	1	−360	360
3	4	0.06701	0.17103	0.0128	9900	0	0	0	0	1	−360	360
4	5	0.01335	0.04211	0	9900	0	0	0	0	1	−360	360
4	7	0	0.20912	0	9900	0	0	0.978	0	1	−360	360
4	9	0	0.55618	0	9900	0	0	0.969	0	1	−360	360
5	6	0	0.25202	0	9900	0	0	0.932	0	1	−360	360
6	11	0.09498	0.1989	0	9900	0	0	0	0	1	−360	360
6	12	0.12291	0.25581	0	9900	0	0	0	0	1	−360	360
6	13	0.06615	0.13027	0	9900	0	0	0	0	1	−360	360
7	8	0	0.17615	0	9900	0	0	0	0	1	−360	360
7	9	0	0.11001	0	9900	0	0	0	0	1	−360	360
9	10	0.03181	0.0845	0	9900	0	0	0	0	1	−360	360
9	14	0.12711	0.27038	0	9900	0	0	0	0	1	−360	360
10	11	0.08205	0.19207	0	9900	0	0	0	0	1	−360	360
12	13	0.22092	0.19988	0	9900	0	0	0	0	1	−360	360
13	14	0.17093	0.34802	0	9900	0	0	0	0	1	−360	360

**Table 3:** Comparison of mean square error of different methods.

Method	MSE
VAR	0.3613
AR	1.5945

## Data Availability

The data used to support the findings of this study are included within the article.
